# Viral Vaccine Adjuvant Strategies for Shaping Durable Immunity Across the Human Lifespan

**DOI:** 10.3390/vaccines14060508

**Published:** 2026-06-04

**Authors:** Swarandeep Singh, Surabhi Gautam, Vidhi Thakkar, Sanjeev Kumar, Devyani Joshi

**Affiliations:** 1 Department of Biochemistry, All India Institute of Medical Sciences, New Delhi 110029, India; swarandeep@aiims.edu; 2 Department of Cell Biology, Emory University School of Medicine, Atlanta, GA 30322, USA; 3 Department of Orthopedics, Emory Musculoskeletal Institute, Emory University School of Medicine, Atlanta, GA 30329, USA; 4 Department of Pharmaceutical Sciences, School of Pharmacy, Massachusetts College of Pharmacy and Health Sciences, Boston, MA 02115, USA; vthak3@stu.mcphs.edu; 5 ICGEB-Emory Vaccine Program, International Centre for Genetic Engineering and Biotechnology, New Delhi 110067, India

**Keywords:** vaccine adjuvants, immunogenicity, innate immunity, systems vaccinology, immunosenescence, infant immunity, nanoparticle adjuvants, CpG oligonucleotides, vaccine durability, precision vaccinology

## Abstract

Vaccination remains one of the most effective strategies for preventing infectious diseases. Yet, the success of modern vaccines increasingly depends on the rational design of adjuvants that enhance and shape immune responses. In this review, we examine current and emerging adjuvant strategies for viral vaccines across the human lifespan. Traditional adjuvants, particularly aluminum salts, have long served as the foundation of vaccine formulations. Still, their limitations have driven the exploration of novel platforms, including emulsions, nucleic acid-based adjuvants, and advanced particulate delivery platforms with intrinsic immunostimulatory properties. These newer approaches act through diverse mechanisms, such as activating innate immune pathways via pattern recognition receptors (PRRs) and stimulating antigen-presenting cells (APCs), thereby improving both humoral and cellular immunity. Recent advances in molecular biology, nanotechnology, and systems vaccinology have deepened mechanistic understanding and enabled more precise modulation of immune responses. However, significant challenges remain, including incomplete knowledge of adjuvant mechanisms, limited diversity among licensed adjuvants, safety concerns, and inconsistent efficacy across age groups. In particular, immune immaturity in infants and immunosenescence in older adults highlight the need for age-specific adjuvant strategies. The review identifies critical gaps in comparative studies, long-term safety data, and the development of adjuvants capable of inducing broad and durable immunity. Further, this article integrates licensed and emerging viral vaccine adjuvants through a lifespan framework. Addressing these limitations through interdisciplinary research and precision-based approaches will be essential for advancing next-generation vaccines and improving global preparedness for emerging infectious diseases.

## 1. Introduction

Durable protection is a central goal in modern vaccinology. Earlier vaccine development and approval processes focused mainly on peak antibody levels after vaccination. These levels were often measured using assays such as hemagglutination inhibition (HAI) or virus-neutralization tests and were treated as primary correlates of protection [1,2]. Evidence from human cohort studies and clinical trials has broadened this view. Research on vaccines for hepatitis B, yellow fever, hepatitis E, and recombinant zoster shows that the persistence and functional quality of immune memory are also critical for long-term protection, particularly several years after immunization [3,4,5,6,7].

Long-lasting immunity depends on several coordinated processes within the adaptive immune system. High-quality antibody responses and memory B cells (MBCs) arise in germinal centers (GCs) within secondary lymphoid tissues [8]. These structures support affinity maturation and selection of B cells with improved antigen binding. Some of these cells later differentiate into long-lived plasma cells (LLPCs) that migrate to the bone marrow and other survival niches [9,10,11]. These plasma cells can continue producing antibodies for many years. Effective long-term immunity also depends on appropriate support from T follicular helper (Tfh) cells and other CD4 T-cell subsets [10,11,12]. Human bone marrow studies show that plasma cells specific for viral and toxoid antigens, such as smallpox, tetanus, and measles, can persist for decades and maintain stable antibody concentrations [5]. By contrast, longitudinal studies of SARS-CoV-2 mRNA vaccines show that circulating neutralizing antibody titers decline within months after primary immunization, even though memory B cells and T cells remain detectable, and direct evidence on the magnitude and stability of long-lived plasma cell responses in humans is still limited [13]. These observations underscore that robust cellular and memory B-cell responses do not always translate into long-lasting circulating antibody levels and highlight the need for better characterization of LLPC formation after newer vaccine platforms.

Host immune status across the lifespan strongly influences both the strength and the durability of vaccine responses. Neonates and young infants have immune systems that differ from those of adults in several ways. Their dendritic cells often produce lower amounts of type I interferons and IL-12 and express fewer co-stimulatory molecules, and neonatal immune responses are increasingly understood as highly regulated and context-dependent; in many settings, they show attenuated Th1-polarizing cytokine production with a relative favoring of Th2-associated or regulatory pathways rather than a uniformly Th2-skewed profile [14,15,16]. Human studies associate these features with weaker Th1-polarizing responses and reduced functional antibody production after some vaccines [17]. Direct histologic information on GC formation in human neonates remains limited. At the opposite end of the age spectrum, older adults experience immunosenescence [18]. This process includes reductions in naïve T-cell and B-cell populations, accumulation of highly differentiated lymphocytes, and functional changes in innate immune cells. Aging is also associated with chronic low-grade inflammation, often described as inflammaging [19]. Together, these factors reduce antigen presentation efficiency, narrow antigen receptor diversity, and contribute to weaker or shorter-lived vaccine responses [18,19,20].

Recognition of these age-related immune differences has changed the understanding and clinical use of vaccine adjuvants. Adjuvants were once viewed mainly as general amplifiers of antibody responses. Current evidence from human immunology studies, transcriptional profiling, and clinical trials shows that they also influence how immune responses are programmed [21,22,23,24]. They can shape the magnitude, quality, and persistence of immunity. Experimental work in humans and animal models indicates that early innate signals influence the differentiation of Tfh cells, the formation of GCs, and the development of LLPCs and MBCs [25,26]. However, many of the detailed mechanistic links have been better characterized in experimental systems than in humans. Achieving long-lasting serologic protection remains challenging. Longitudinal studies in humans show that high antibody levels shortly after vaccination do not always lead to durable immunity [13]. This is particularly evident when LLPCs are not effectively established in the bone marrow [27]. Recent systems vaccinology research has begun to identify early biological signals that correlate with long-term antibody persistence [21,22,23].

This review examines durable viral vaccine immunity across the human lifespan by integrating mechanistic insights with clinical evidence from licensed vaccines. Major classes of adjuvants are considered, including aluminum salts, squalene-based emulsions such as MF59 and AS03, CpG-containing formulations, and saponin-based systems such as AS01. Particular attention is given to how these adjuvants influence innate immune activation, Tfh cell responses, GC biology, and LLPC generation. The discussion also addresses how these mechanisms operate in different populations, including children, adults, pregnant individuals, and older adults. Understanding these relationships will be essential for optimizing adjuvant strategies that support durable protection in contemporary viral vaccine development.

## 2. Age-Dependent Immune Landscapes, Correlates of Durable Protection, and Adjuvant Strategies Across the Lifespan

**Figure 1:**
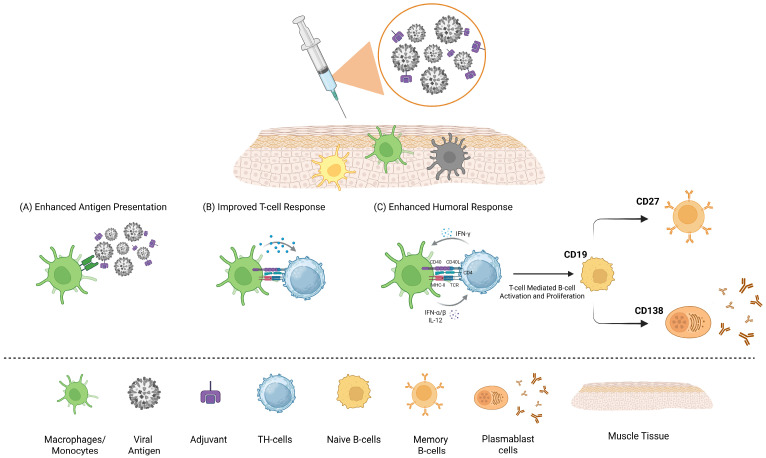
Adjuvant-mediated enhancement of innate and adaptive immune responses. (**A**) Enhanced antigen uptake and presentation by innate immune cells, including macrophages and monocytes, leading to improved antigen processing and presentation via major histocompatibility complex (MHC) molecules. (**B**) Robust T-cell responses, characterized by increased activation, proliferation, and differentiation of antigen-specific CD4^+^ and CD8^+^ T cells. (**C**) Enhanced humoral immunity through T cell–dependent B cell activation, resulting in B cell proliferation and differentiation into memory B cells and antibody-secreting plasmablasts. Created in https://BioRender.com.

Durable antiviral immunity depends on the coordinated persistence of several immune components, including neutralizing antibodies (nAbs), LLPCs, MBCs, memory T lymphocytes, and innate immune programs that shape adaptive responses [26,28,29,30,31]. In humans, plasma cells specific for vaccines such as measles, tetanus, and varicella have been detected in bone marrow decades after immunization, and their presence correlates with stable antibody concentrations in the blood [5,32,33,34]. By contrast, vaccines that do not efficiently generate LLPCs may show faster declines in circulating antibodies. This pattern has been observed after vaccination with the mRNA COVID-19 vaccines, including Comirnaty and Spikevax. In these cases, nAb levels decline within months, even though MBCs and T cells remain detectable [13,35]. Studies of measles vaccination also show that antibody levels can decrease gradually in elimination settings, particularly among children vaccinated very early in life [36]. These findings indicate that durable antibody protection depends on the quality of GC responses, formation of LLPCs, and the age at first vaccination rather than on peak antibody levels alone. Because the immune system changes substantially across the lifespan, from the tightly regulated environment of the neonate to the inflammaging milieu of the older adult, vaccine strategies that account for these biological differences can meaningfully improve both the magnitude and durability of protection (Figure 1).

### 2.1. Neonates (Under 1 Month)

Immune responses in newborns develop in a tightly regulated environment that helps prevent excessive inflammation early in life. Neonatal cord blood immune cells express PRRs, including TLR2, TLR4, TLR8, and TLR9, yet dendritic cells often produce lower levels of IL-12p70, tumor necrosis factor alpha, and type I interferons after TLR stimulation, while producing higher levels of IL-10 [37,38,39,40]. These differences reflect changes in intracellular signaling and gene transcription rather than the absence of receptors. This pattern often leads to weaker Th1 polarization and reduced interferon gamma responses after primary vaccination. It can limit recall responses to certain vaccines, although others, such as BCG and measles vaccines, can generate strong recall responses after boosting [41,42,43,44]. Neonatal antigen-presenting cells also exhibit differences in signaling pathways and costimulatory molecule expression, which can limit T cell activation and antibody responses [45]. Direct measurements of GC and Tfh cell activity in newborn humans remain limited.

Laboratory studies using cord blood immune cells show that combinations of TLR agonists can partly overcome these limitations. For example, combined stimulation of TLR3, TLR4, and TLR7 or TLR8 can increase production of IL-12p70, interferon gamma, and interferon alpha, producing cytokine patterns that resemble adult responses [17]. Experimental work suggests that certain cytosolic PRR agonists, including double-stranded RNA mimetics and cytosolic dinucleotides, may overcome some limitations of neonatal TLR signaling. However, most supporting evidence comes from experimental models and ex vivo studies [46,47].

In clinical settings, vaccines that possess strong intrinsic immune stimulation, such as live attenuated or whole-cell vaccines, such as the Bacille Calmette–Guérin (BCG) vaccine, can induce robust Th1 responses at birth [48,49]. These findings indicate that neonatal vaccine responses can be improved by adjuvants that stimulate antiviral innate pathways while maintaining acceptable safety.

### 2.2. Infants and Toddlers (1 to 36 Months)

During infancy and early childhood, immune function matures rapidly. During the first year of life, TLR responses and cytokine production gradually become more similar to adult patterns, which coincides with improved responses to vaccination [45]. Dendritic cell activity, T cell differentiation, and B cell repertoires develop rapidly, leading to stronger antibody responses to vaccines. However, for certain vaccines given very early in life, such as measles vaccines administered in early infancy, antibody levels may decline faster than when vaccination occurs at an older age, reflecting incomplete development of durable LLPC responses at the time of priming [36,50,51].

Influenza vaccines containing the MF59 adjuvant are immunogenic and generally well tolerated in infants and young children, producing stronger antibody responses than non-adjuvanted formulations [52,53]. Adjuvanted influenza vaccines have shown promise in this age group. Vaccines formulated with MF59 or AS03 can increase hemagglutination inhibition antibody titers and improve cross-reactive responses against different influenza strains [53,54]. Some clinical studies also report improved protection against influenza illness in young children with acceptable safety profiles [55,56,57]. These adjuvants recruit antigen-presenting cells to the injection site and enhance antigen uptake and T follicular helper cell differentiation, which likely support GC activity and the development of durable antibody responses. However, direct measurements in human lymphoid tissue remain limited [52,58].

### 2.3. Pregnancy

Pregnancy involves coordinated immune adjustments that allow the maternal immune system to tolerate the fetus while maintaining defense against infections. This state includes increased regulatory T cell activity, changes in cytokine signaling, and modifications in antibody structure and function [59,60,61]. Maternal vaccination takes advantage of this biology because immunoglobulin G antibodies generated during pregnancy can cross the placenta via the neonatal Fc receptor [62,63]. The efficiency of this transfer depends on antibody subclass and Fc glycosylation patterns, which also influence antibody activity in newborns.

Vaccines against influenza, tetanus, diphtheria, and pertussis are widely recommended during pregnancy. These vaccines have demonstrated strong safety profiles and protect both the mother and the infant, largely through antibodies transferred before birth [64]. Most of these vaccines use aluminum salt adjuvants or contain no additional adjuvant. Aluminum salts have extensive safety data in pregnancy and reliably induce antibody responses that are efficiently transferred to the fetus.

More recently, maternal vaccination strategies have expanded to include the prevention of respiratory syncytial virus. The prefusion F protein RSV vaccine, Abrysvo, has shown substantial protection against medically attended RSV lower respiratory tract disease in infants younger than six months when administered late in pregnancy [65,66,67,68]. Systems immunology studies suggest that differences in antibody glycosylation and subclass composition can affect the efficiency with which antibodies cross the placenta and function in the newborn [63,69]. These observations may guide future design of maternal vaccines and their adjuvants. More potent adjuvants, such as AS01 or MF59, are currently being evaluated in maternal vaccine research [70]. Because maternal vaccines require very high safety standards, most licensed vaccines in pregnancy continue to rely on adjuvants with the longest safety records while clinical trials assess newer formulations.

### 2.4. Older Adults (Over 65 Years)

In older adults, immune aging leads to several functional changes. The number of naïve T and B cells declines, senescent or exhausted T cells accumulate, and antigen-presenting cell activity becomes less efficient [18,20]. Cytokine signaling pathways may also shift toward chronic low-level inflammation. Chronic low-grade inflammation further alters monocyte and dendritic cell responses [19]. These factors contribute to weaker vaccine responses and faster antibody decline in older adults.

Adjuvanted vaccines can partially compensate for these limitations. The recombinant zoster vaccine Shingrix uses the AS01B adjuvant system and elicits strong CD4 T cell responses and high antibody levels, even in adults aged 70 years and older. Long-term follow-up studies show sustained protection against herpes zoster for at least a decade after vaccination [3]. Emulsion-based adjuvants such as MF59 and AS03, as well as saponin and MPLA combinations such as AS01, enhance innate immune activation, improve CD4 T cell responses, increase antibody titers, and, in some cases, extend the duration of protection compared with non-adjuvanted vaccines [24,25,71,72]. Influenza vaccines formulated with MF59 or AS03 have demonstrated modest but consistent improvements in antibody responses and vaccine effectiveness compared with standard dose non-adjuvanted influenza vaccines in older adults. Several national immunization programs now recommend these vaccines for older populations [58,73].

Researchers are also exploring whether some vaccines can stimulate “trained immunity,” a process in which innate immune cells undergo epigenetic changes that enhance responses to subsequent infections [74,75,76]. Studies of the BCG vaccine in adults, including older health care workers, have shown changes in DNA methylation patterns and stronger responses to COVID-19 vaccines [77]. However, current evidence does not clearly demonstrate broad protection against unrelated respiratory infections or reductions in mortality.

To integrate the age-related considerations, Table 1 summarizes main vaccines administered at different stages of life according to the standard immunization schedules, and their corresponding adjuvants.

## 3. Licensed and Late-Stage Adjuvanted Viral Vaccines

**Figure 2:**
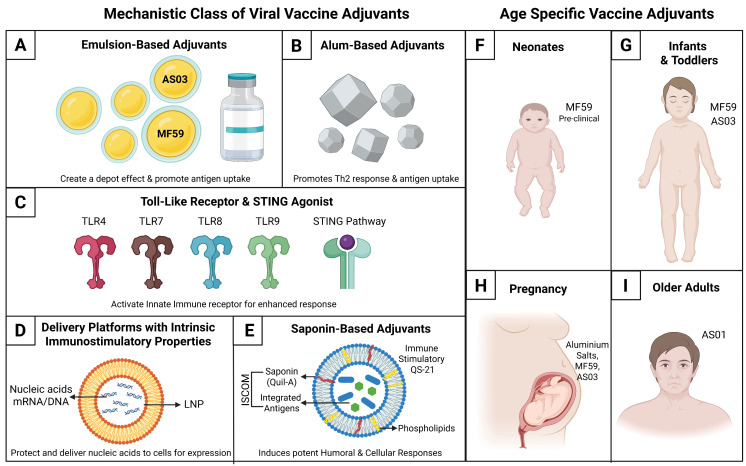
(**A**–**E**) Major mechanistic classes of viral vaccine adjuvants. (**A**) Emulsion-based adjuvants (e.g., MF59, AS03) that enhance immune cell recruitment and antigen uptake. (**B**) Alum-based adjuvants (aluminum hydroxide or phosphate) that promote antigen depot formation and Th2-biased humoral responses. (**C**) Pattern recognition receptor (PRR) agonists, including Toll-like receptor (TLR4, TLR7/8, TLR9) and stimulator of interferon genes (STING) agonists, which activate innate immune signaling pathways and cytokine production. (**D**) Delivery platforms and self-adjuvanting nucleic acid formulations, such as lipid nanoparticles (LNPs) and poly (lactic-co-glycolic acid) (PLGA), that facilitate antigen delivery and enhance antigen presentation. (**E**) Saponin-based adjuvants (e.g., AS01) that promote robust cellular and humoral immune responses, including CD8^+^ T cell activation. (**F**–**I**) Age-specific considerations in the selection of vaccine adjuvants. (**F**) Emulsion-based adjuvants (e.g., MF59) in early life to enhance immunogenicity in neonates. (**G**) Emulsion-based systems (MF59, AS03) in infants and toddlers (from 6 months of age) to improve the magnitude and breadth of immune responses. (**H**) Alum-based adjuvants during pregnancy due to their established safety profile and ability to induce protective antibody responses. (**I**) Saponin-based adjuvant systems (e.g., AS01) in older adults to overcome immunosenescence and enhance both cellular and humoral immunity. Created in https://BioRender.com.

Licensed adjuvanted viral vaccines provide a useful way to understand how adjuvants shape the strength and persistence of immune responses in humans (Figure 2). This is especially important at the extremes of age, such as in young children and older adults. Evidence from large randomized trials and real-world studies shows that adjuvants that strongly stimulate innate immune pathways and enhance CD4 T cell support can increase vaccine protection and, in some cases, prolong immunity compared with vaccines that lack adjuvants or use weaker ones [78,79].

The recombinant zoster vaccine, Shingrix (RZV), provides a clear example of improved durability in older adults who experience age-related immune decline [80]. This vaccine contains the varicella zoster virus (VZV) glycoprotein E, combined with the AS01B adjuvant system, which includes MPLA and QS-21 in liposomes [18]. In the phase 3 trials, the ZOE-50 and the ZOE-70 trials, vaccine efficacy reached 97.2 percent in adults aged 50 years or older and about 90 percent in adults aged 70 years or older against herpes zoster [81,82]. Median follow-up in these trials ranged from about three to nearly four years. Long-term follow-up in the ZOE-LTFU study showed that protection remains high for up to 10 years after vaccination [83]. Overall vaccine efficacy was 81.6 percent between years 5.6 and 9.6, with yearly estimates above 80 percent through year eight and around 73 percent by years nine to ten. During this period, antibodies directed against glycoprotein E and polyfunctional CD4 T cell responses remain more than five to six times higher than baseline. These findings offer strong human evidence that a potent adjuvant can sustain long-term protection in older adults.

Adjuvanted influenza vaccines provide another example of how strengthening innate and CD4 T cell responses can improve vaccine performance in older adults, even though antibody levels still decline within a year. The MF59-adjuvanted influenza vaccine, Fluad, was developed to counteract the effects of immune aging [84]. Observational studies and comparative effectiveness analyses indicate that this vaccine offers modest but consistent improvements in protection against cardiorespiratory hospitalizations and laboratory-confirmed influenza compared with standard-dose influenza vaccines without adjuvants. Its performance is generally comparable to that of high-dose egg-based influenza vaccines in adults aged 65 years or older. During the 2009 H1N1 influenza pandemic, vaccines containing the AS03 adjuvant also achieved high seroconversion rates while requiring less antigen [85]. Randomized trials showed that protective antibody levels persisted for at least 6 to 12 months in both younger and older adults [86,87]. MBCs and CD4 T cell responses remained detectable beyond the first influenza season. Although influenza antibody titers usually decline within a year across all age groups, adjuvanted formulations often maintain higher antibody levels and higher seroprotection rates than non-adjuvanted vaccines over the same period. This supports their use in populations with a high risk of severe disease.

The CpG 1018 adjuvanted hepatitis B vaccine, HEPLISAV-B, uses a TLR-9 agonist to drive a Th1-biased immune response. In phase 3 randomized trials involving adults, HEPLISAV-B achieved faster and higher rates of seroprotection against hepatitis B surface antigen than the three-dose alum-adjuvanted vaccine ENGERIX-B [88,89]. Protection was defined as an anti-HBs antibody concentration of at least 10 IU/L. These benefits were observed across different age groups and risk categories. Retrospective observational studies in people living with HIV and other high-risk groups also show higher seroconversion rates [90]. In many recipients, protective antibody levels remain above the threshold for several years, although long-term direct comparisons of durability remain limited. Mechanistic studies show that CpG 1018 activates plasmacytoid dendritic cells and promotes interferon-associated cytokines and Th1 responses [91]. These immune signals likely support the formation of MBCs and LLPCs.

Matrix-M is a saponin-based nanoparticle adjuvant used in the recombinant spike protein SARS-CoV-2 vaccine, Novavax COVID-19 Vaccine [91]. Phase 3 trials in adults and adolescents demonstrated strong protection against symptomatic COVID-19 and robust nAb and MBC responses after a two-dose schedule [92]. Safety outcomes were also favorable. Follow-up studies and real-world data show that, similar to other COVID-19 vaccines, protection against infection and mild disease declines over time [93]. Protection against severe disease remains more stable but still requires booster doses, especially in older adults and people at higher risk [94,95].

In contrast, the RSV vaccines now approved for older adults are Arexvy, Abrysvo and mRESVIA. Arexvy uses a stabilized prefusion F protein combined with the AS01E adjuvant system, whereas Abrysvo is a non-adjuvanted stabilized prefusion F protein vaccine and mRESVIA is an mRNA vaccine encoding prefusion F protein. Phase 3 trials in adults aged 60 years or older showed strong protection against RSV-associated lower respiratory tract disease during the first season after vaccination [96]. A large target trial emulation conducted among United States veterans found that vaccine effectiveness against documented Respiratory syncytial virus infection declined over time [96]. Effectiveness decreased from roughly 82 to 83 percent during the first month after vaccination to about 59.4 percent during follow-up of up to 18 months. The decline was more pronounced in immunocompromised individuals. These findings show that even potent, highly immunogenic vaccines can experience substantial reductions in effectiveness within 1 to 2 seasons, particularly in vulnerable populations.

To facilitate comparison across the main vaccine adjuvants discussed above, Table 2 summarizes the viral vaccine adjuvants and their key advantages and limitations.

## 4. Mechanistic Classes of Viral Vaccine Adjuvants and Their Interplay with Vaccine Platforms in Shaping Immune Durability

Viral vaccine adjuvants can be organized into mechanistic classes according to how they activate innate immune pathways and shape downstream adaptive responses. These signals affect GC formation, T cell polarization, antibody isotype distribution, and, in some settings, the development of tissue-resident immune memory. Vaccine outcomes still depend on the antigen, the vaccine platform, and the host immune state. Even so, adjuvant selection strongly influences the magnitude and quality of immune responses and can affect the persistence of immune memory in humans. The vaccine platform-specific properties interact with adjuvant activity to determine the magnitude, quality, and persistence of immune memory in humans.

Adjuvant selection is therefore tightly linked to both the vaccine platform and the primary immunological goal. For inactivated and split-virus influenza vaccines that must be reformulated and administered repeatedly because of antigenic drift, oil-in-water emulsions such as MF59 or AS03 are chosen to compensate for the lower intrinsic immunostimulatory capacity of the platform and to broaden and boost antibody responses across seasons, while maintaining an acceptable reactogenicity profile for annual revaccination [97]. In contrast, for recombinant protein or VLP vaccines where the goal is to generate high-titer, durable, and often Th1-biased responses, adjuvants such as AS01 or CpG-containing systems are prioritized to drive strong T follicular helper cell support, germinal center formation, and long-lived plasma cell and memory B cell responses [103]. Nucleic acid and viral-vector vaccines incorporate self-adjuvanting properties, so additional adjuvants are generally not added; instead, formulation and dosing strategies are tuned to balance strong, rapidly induced cellular and humoral immunity against the risk of excessive inflammation, especially when booster doses are required. Together, these examples illustrate that platform-specific constraints, the frequency of booster administration, and the desired balance between breadth, magnitude, and durability of immunity all shape rational adjuvant selection in practical vaccine design. The subsections below address each adjuvant class, along with the vaccine platforms in which it is most commonly employed.

### 4.1. Emulsion-Based Adjuvants

Oil-in-water emulsions such as MF59 and AS03 are among the most widely used adjuvants in licensed influenza vaccines and are most commonly applied to inactivated and split-virus platforms. Chemical or heat inactivation abolishes replication and reduces PAMP signals relative to live-attenuated or replicating viral-vector vaccines; as a result, inactivated vaccines often elicit lower peak neutralizing antibody titers and less robust CD8^+^ T cell responses without adjuvantation, particularly in older adults, although protective humoral immunity and measurable T cell responses are still induced in many populations. Human immunology and systems vaccinology studies show that MF59 addresses this limitation by enhancing early innate activation at the injection site. It promotes the recruitment of monocytes and other antigen-presenting cells (APCs), increases antigen uptake and trafficking to draining lymph nodes, and strengthens GC B cell and Tfh cell responses as demonstrated in animal models and inferred from analogous processes in humans. Clinical studies in adults and children consistently show that MF59 or AS03 adjuvanted influenza vaccines produce higher HAI antibody titers, broader strain coverage, and in some age groups, more sustained antibody responses than non-adjuvanted vaccines [52,54,58].

In clinical practice, MF59-adjuvanted influenza vaccines provide modest but consistent improvements in protection against influenza and related hospitalizations in older adults compared with standard-dose non-adjuvanted vaccines [73,104]. Their effectiveness is generally comparable to that of high-dose influenza vaccines in several influenza seasons. For inactivated SARS-CoV-2 vaccines such as CoronaVac, phase 1/2 and post-licensure studies show robust initial antibody responses in many adults, with lower titers in older and immunocompromised populations, and substantial waning of neutralizing antibodies and protection against infection over 3–6 months. Nevertheless, protection against severe disease is better maintained, especially when homologous or heterologous boosters are administered [105,106]. These data underscore the importance of booster or mixed-platform strategies for durable protection against infection. Whole-virion formulations may preserve a broader set of epitopes than split vaccines, which could theoretically support broader B- and T-cell recognition, but direct human evidence that whole-virion inactivated vaccines yield substantially more durable protection than split formulations remains limited.

In studies of adults who received vaccines formulated with the AS03 adjuvant and other platforms, a blood transcriptional signature related to platelet activity and cell adhesion, measured about 7 days after vaccination, predicted the durability of antibody responses [21,22,23]. This pattern was observed across several vaccines, including influenza, COVID-19 mRNA vaccines, meningococcal vaccines, malaria vaccines, and pneumococcal conjugate vaccines [13,21,22,23]. Follow-up experiments suggested that megakaryocytes activated by thrombopoietin can support the survival of human bone marrow plasma cells through integrin-mediated interactions and the production of survival factors, including APRIL and macrophage migration inhibitory factor. These findings indicate that platelet and megakaryocyte pathways may contribute to plasma cell survival niches alongside traditional stromal cell support [21]. Although such biomarkers are not yet used routinely in clinical practice, they represent an important step toward identifying measurable predictors of durable vaccine immunity.

### 4.2. Alum-Based Adjuvants

Aluminum salts remain the most widely used adjuvants in vaccines and have a long safety record across diverse populations, including children and pregnant women. Alum forms particulate complexes with antigens that promote uptake by APCs [45,107]. Laboratory studies show that alum can activate inflammasome pathways and stimulate the release of host-derived danger signals such as extracellular DNA. These signals promote the production of cytokines, including IL-1β and IL-18, and create an environment that supports strong antibody responses, which often display a Th2 bias [108].

In clinical use, alum-adjuvanted hepatitis B vaccines reliably generate protective antibody responses in most healthy adults and children [45,109]. However, individuals with conditions such as chronic kidney disease or HIV infection often show weaker responses and may require additional doses or alternative formulations [90]. Alum is less efficient at generating strong CD8 T cell responses or highly polarized Th1 cellular immunity. For this reason, it is often combined with other immunostimulatory components such as TLR agonists in newer adjuvant systems. In recombinant protein and VLP-based vaccines, alum-adjuvanted hepatitis B and HPV vaccines have demonstrated excellent long-term humoral immunogenicity, with high seropositivity and strong protection against HPV-related lesions maintained for 10–12 years after immunization in adolescents and young children, illustrating that appropriate scheduling and formulation can achieve durable antibody responses even with Th2-skewed adjuvants [79,110,111].

### 4.3. Toll-like Receptor and Sting Agonists in Recombinant Protein and Vlp Vaccines

Adjuvants that directly activate TLRs represent another mechanistic class that promotes Th1-oriented immunity and stronger T cell help. Licensed examples include MPLA, a TLR-4 agonist contained in the AS01 and AS04 system, and CpG 1018, a TLR-9 agonist used in the hepatitis B vaccine Heplisav-B [90,112]. These molecules trigger innate signaling cascades that lead to the production of type I interferons, IL-12, and related cytokines. The resulting environment supports maturation of APCs, expansion of Tfh cells, and GC reactions that produce high-affinity antibodies and durable MBCs.

Recombinant protein and VLP vaccines have low intrinsic immunogenicity and therefore depend substantially on adjuvants of this class to induce strong GC responses and durable memory. The AS01 adjuvant system, containing MPLA and the saponin QS-21 in a liposomal formulation, illustrates this. Clinical trials of the AS01B adjuvanted recombinant zoster vaccine show strong polyfunctional CD4 T cell responses and sustained antibody levels in older adults [3]. In the RTS, S/AS01 malaria vaccine, AS01 supports robust humoral and cellular immunity, conferring significant protection. Systems vaccinology studies of hepatitis B vaccines formulated with AS01 or AS03 have identified early interferon-related transcriptional signatures that correlate with later antibody magnitude and function. These findings suggest that early innate activation patterns may influence the long-term characteristics of vaccine-induced immunity. The CpG 1018 adjuvanted hepatitis B vaccine produces higher and faster seroprotection rates than alum-based comparators across several adult populations [90], further showing an advantage of Th1-skewing TLR agonists in this platform.

Matrix-M, a saponin-based nanoparticle adjuvant, enhances vaccine responses in NVX-CoV2373 and other protein-based vaccines by promoting antigen delivery to draining lymph nodes and increasing the recruitment and activation of antigen-presenting cells [101]. Human phase 3 and booster trials demonstrate that Matrix-M adjuvanted NVX-CoV2373 elicits high neutralizing titers and broad, though variant-dependent, neutralization against multiple SARS-CoV-2 variants, translating into substantial effectiveness against symptomatic and severe COVID-19, with waning over time similar to other platforms and responsiveness to boosting [113].

Stimulator of interferon gene (STING) agonists are also being investigated as vaccine adjuvants. Preclinical studies demonstrate potent induction of CD8 T cell responses and strong antitumor activity [114]. However, translation to human vaccines has been challenging. Differences in STING signaling between species and delivery challenges have slowed clinical progress [115]. New prodrug and nanoparticle formulations are under development, but currently, no STING-based adjuvant has been widely adopted for licensed viral vaccines.

### 4.4. Nucleic Acid Vaccines and Lipid Nanoparticle Delivery Platforms with Intrinsic Immunostimulatory Properties

Modern mRNA vaccines use RNA cargo to encode antigen, while lipid nanoparticles (LNPs) primarily serve as delivery systems that protect nucleic acids and facilitate cellular uptake. Depending on formulation composition, both the RNA and lipid components can also contribute intrinsic innate immune stimulation; therefore, these systems are best described as delivery platforms with self-adjuvanting properties rather than classical adjuvants [116].

LNP-encapsulated mRNA vaccines against SARS-CoV-2 illustrate this concept [117,118]. LNPs protect mRNA and facilitate cellular uptake, where translated antigen drives B- and T-cell priming. In humans, both the mRNA and the LNP carrier activate innate sensors, including TLR-7 and 8, and cytosolic receptors such as RIG-I and MDA5 [119,120]. These pathways induce type I interferons and inflammatory cytokines that drive antigen-presenting cell maturation and strong CD4 and CD8 T cell responses, although the exact contributions of individual RNA sensors (TLR7, TLR8, RIG-I, MDA5) are inferred largely from non-clinical data.

mRNA COVID-19 vaccines generate strong GC activity in draining lymph nodes and elicit robust nAb and MBC responses [28,102]. Longitudinal cohort studies demonstrate that nAb levels typically decline within 3 to 6 months after 2 doses, with further waning after a third dose, especially against immune-evasive variants such as Omicron [121]. Booster doses, whether homologous mRNA or heterologous vector or protein vaccines, restore antibody titers and broaden variant coverage. Some heterologous booster trials using adjuvanted protein vaccines after mRNA priming show non-inferior or superior neutralization against selected variants compared with additional mRNA doses, but long-term comparative durability data are still emerging [122]. Protection against severe disease tends to persist longer because MBCs and T cell responses remain active even as antibody levels wane.

Studies of bone marrow samples indicate that SARS-CoV-2-specific ASCs are present but may be less stably represented in LLPC compartments than those generated by some long-established vaccines, such as tetanus [13,123]. Direct comparisons of LLPC formation across mRNA and protein-based vaccines remain limited, and conclusions about durability should therefore be interpreted cautiously. Self-amplifying RNA platforms and next-generation LNPs are in early clinical development and aim to improve dose-sparing and durability, but robust human evidence for superior long-term immunity compared with first-generation mRNA vaccines is not yet available.

### 4.5. Viral Vector Vaccines

Non-replicating viral vector vaccines, such as adenoviral vectors and modified vaccinia Ankara (MVA), retain key features of viral infection that promote strong innate activation, antigen presentation, and robust CD4^+^ and CD8^+^ T-cell responses without the need for exogenous adjuvants. Adenoviral vector vaccines against Ebola and COVID-19 (e.g., Ad26.ZEBOV, ChAdOx1-nCoV-19, Ad26.COV2.S) consistently induce polyfunctional CD8^+^ T-cell responses and protective antibody titers in phase 1–3 trials and real-world studies [124].

Heterologous prime–boost strategies combining different viral vectors or vector priming followed by protein or mRNA boosting can further enhance the breadth and durability of both antibody and T-cell responses. HIV vaccine trials using Ad26 and MVA vectors, as well as vector plus Env protein regimens, have reported durable CD8^+^ and CD4^+^ responses lasting 1–2 years, although clinical efficacy against HIV acquisition remains limited [125]. These data support the concept that viral vectors provide a strong foundation for cellular immunity, which can be modulated and extended by carefully designed boosting regimens, including heterologous platforms that incorporate adjuvanted protein components.

### 4.6. Rational Adjuvant Combinations

Many modern adjuvant systems combine several innate stimuli to improve immune responses. The AS01 system is a well-known example. It contains MPLA, a TLR-4 agonist, together with the saponin QS-21 in a liposomal formulation. This combination activates multiple innate pathways and produces strong Th1-biased CD4 T cell responses along with high antibody titers and durable protection in older adults [3].

Further, CpG oligodeoxynucleotides can be paired with alum to strengthen Th1-oriented antibody responses and accelerate the development of protective antibody levels compared with alum alone. Some observational studies suggest that these strategies may also improve the persistence of antibody responses in certain populations, although very long term follow up data are still accumulating. Systems vaccinology studies of hepatitis B vaccines formulated with AS01 or AS03 have identified early interferon-related transcriptional signatures that correlate with later antibody magnitude and function [90]. These findings suggest that early innate activation patterns may influence the long-term characteristics of vaccine-induced immunity. Overall, these examples show how combinations of complementary innate signals can generate stronger peak responses and, in some cases, more sustained immune protection. In all cases, appropriate adjuvantation is important for maximizing GC responses and immune persistence.

### 4.7. Next-Generation and Emerging Adjuvant Strategies

Beyond the relatively small set of adjuvants included in licensed viral vaccines, several next-generation approaches are under active investigation in preclinical and early-phase clinical studies. These platforms often combine targeted innate sensing with advanced delivery technologies and aim not only to increase the magnitude of responses, but also to broaden epitope coverage, improve durability, or more precisely shape the quality of immunity in specific age groups [126]. One area of interest involves rationally designed nanoparticle and self-assembling protein platforms that incorporate intrinsic immunostimulatory motifs or co-deliver pattern-recognition receptor agonists. Compared with alum or classical oil-in-water emulsions, these systems can localize antigens and adjuvants to draining lymph nodes, promote sustained germinal center activity, and favor the generation of broadly cross-reactive memory B cells and long-lived plasma cells [127]. Age-focused studies in neonatal and aged animal models suggest that nanoparticle formulations containing TLR7/8 or STING agonists can partially overcome impaired Th1-polarizing cytokine production and support more durable antibody and T-cell responses than traditional formulations, although human data remain limited [128].

A second emerging strategy is the use of systems vaccinology and high-dimensional immune profiling to guide adjuvant selection and formulation. Early transcriptional and cellular signatures after vaccination have been linked to downstream antibody persistence for several platforms, and these data are being used prospectively to design adjuvants that preferentially induce durability-associated innate programs rather than relying solely on peak titer readouts [129,130]. Such approaches differ from historical adjuvant development, which was largely empirical, and may enable precision adjuvantation tailored to age, baseline immune phenotype, or comorbid conditions.

Finally, there is growing interest in adjuvant systems that intentionally modulate tissue-resident immunity or trained immunity pathways, including formulations that target skin, respiratory mucosa, or bone marrow niches. These strategies seek to extend protection beyond conventional circulating neutralizing antibodies by enhancing local memory, cross-protective responses against variant or heterologous viruses, and resilience in populations with immune immaturity or immunosenescence [131]. While most of these concepts are supported primarily by experimental models, they illustrate how next-generation adjuvants may eventually complement or replace traditional alum- and emulsion-based adjuvants in age-specific vaccine programs.

## 5. Safety, Reactogenicity, and Public Confidence in Adjuvanted Viral Vaccines Across Age Groups

Adjuvanted viral vaccines have been studied in large randomized clinical trials and in extensive post-marketing safety monitoring programs. Together, these data provide strong evidence that these vaccines have acceptable safety and tolerability across age groups [57,132,133]. Licensed adjuvants such as alum, MF59, AS03, AS04, and AS01 are mainly associated with short-lived local and systemic reactions. Serious vaccine-related adverse events are uncommon and usually occur at rates similar to those seen in control groups. Patterns of reactogenicity vary according to the type of adjuvant, the antigen used, and host factors. These differences are considered during vaccine design and dose selection. For the AS01-adjuvanted recombinant zoster vaccine, observational studies in Medicare populations and regulatory reviews have identified a small increase in the risk of Guillain–Barré syndrome within 42 days of vaccination [134,135]. The increase corresponds to only a few additional cases per million doses. Although this is a rare event, it requires clear communication while recognizing that the overall balance of benefits and risks remains favorable.

### 5.1. Reactogenicity Profiles by Adjuvant Class

Different vaccine adjuvants produce characteristic patterns of reactogenicity that reflect how they stimulate the innate immune system. MF59 and AS03 are among the most widely studied adjuvants in licensed viral vaccines, particularly in seasonal and pandemic influenza vaccines. Randomized trials in adults and older adults show that influenza vaccines containing MF59 or AS03 commonly cause local reactions, such as injection-site pain, redness, or swelling, in about 40 to 70 percent of recipients [136,137]. Systemic symptoms, including fatigue, muscle aches, headache, or mild fever, occur in roughly 20 to 40 percent of recipients. These reactions usually begin within one to two days after vaccination and resolve on their own within several days. Pediatric trials of MF59-adjuvanted influenza vaccines show similar patterns [138]. Local reactions are somewhat more frequent, but overall tolerability remains acceptable. Adjuvant systems containing AS01, AS04, or Matrix-M tend to elicit stronger, but still temporary, systemic reactions compared with vaccines containing only alum. These reactions may include higher rates of fever, muscle pain, and general malaise [139,140]. In phase 3 trials of the AS01-adjuvanted recombinant zoster vaccine, grade 3 local or systemic reactions occurred in a noticeable portion of participants. Despite this, rates of serious adverse events and vaccine discontinuation were similar in the vaccine and comparator groups, and the vaccine demonstrated high efficacy against herpes zoster [141]. Overall evidence indicates that adjuvanted viral vaccines may cause more short-term reactions than many non-adjuvanted formulations, but their safety profiles remain predictable and manageable in routine clinical use.

### 5.2. Age-Specific Considerations

Safety considerations for adjuvanted vaccines differ across the lifespan because the immune system changes with age. In infants and young children, adjuvanted formulations are tested in age-specific dose-finding and immunogenicity trials to balance reactogenicity with adequate protection. MF59-adjuvanted influenza vaccines in children produce stronger antibody responses and somewhat higher rates of local reactions compared with non-adjuvanted vaccines. However, serious vaccine-related adverse events are rare, and overall tolerability is considered acceptable [142]. For hepatitis B vaccination in children, most programs continue to use alum-adjuvanted vaccines. These vaccines achieve seroprotection rates above 90 percent and have well-established safety records. Vaccines that use CpG adjuvants for hepatitis B are licensed and well-studied in adults, but published data in pediatric populations remain limited [143].

Pregnancy represents a distinct physiologic and immunologic context in which both maternal and fetal outcomes must be evaluated. Observational cohort studies and pregnancy registries from the 2009 H1N1 influenza pandemic and later seasons show that adjuvanted influenza vaccines containing AS03 or MF59 do not increase the risk of adverse pregnancy or neonatal outcomes. These vaccines also provide effective protection against influenza infection. Maternal vaccination leads to transfer of influenza-specific immunoglobulin G across the placenta. Antibody levels in cord blood are often slightly higher than maternal levels, with cord-to-maternal ratios typically ranging from about 1.2 to 1.7 [144]. This supports protection of newborns during the first months of life. Current evidence therefore supports the use of selected adjuvanted influenza vaccines during pregnancy when recommended, although additional pregnancy-specific data are still needed for some other adjuvanted vaccine platforms.

In older adults, age-related immune changes and chronic low-grade inflammation influence both vaccine responses and the experience of side effects. Randomized trials and secondary analyses show that adjuvanted influenza and zoster vaccines produce stronger neutralizing antibody responses and more robust T-cell responses than non-adjuvanted vaccines in this age group. These enhanced responses contribute to improved protection against severe influenza and herpes zoster. Short-term symptoms such as fatigue, muscle pain, or injection-site discomfort may occur slightly more often or last somewhat longer in older adults [98,141]. However, serious vaccine-related adverse events remain uncommon and occur at frequencies similar to those observed in control groups.

### 5.3. Pharmacovigilance and Regulatory Oversight

After licensure, the long-term safety of adjuvanted vaccines is monitored through national and international pharmacovigilance systems. These include passive reporting systems and active surveillance programs, as well as large observational studies. For adjuvanted vaccines used against human papillomavirus, hepatitis B, and influenza, systematic reviews and meta-analyses that include hundreds of thousands or even millions of vaccine recipients have not identified a substantial increase in autoimmune diseases or chronic inflammatory conditions compared with expected background rates [99]. Some studies have reported small or statistically uncertain increases in specific autoimmune outcomes, but the findings are inconsistent and the absolute risks remain very low. Rare syndromes proposed to be associated with adjuvants, such as autoimmune or inflammatory syndrome attributed to adjuvants, have mainly been described in small case series. Large epidemiologic investigations with appropriate controls have not confirmed this syndrome as a distinct clinical condition. Current population-level evidence therefore suggests that any autoimmune risk related to licensed adjuvanted vaccines is small when compared with the health risks of the infections these vaccines prevent [100,145]. Regulatory agencies such as the U.S. Food and Drug Administration (US-FDA) and the European Medicines Agency continue to evaluate safety signals using advisory committees and updated surveillance data. For example, the small increase in the risk of Guillain–Barré syndrome observed after recombinant zoster vaccination led to updated safety information in product labeling [134]. At the same time, regulatory authorities maintained recommendations for vaccination in older adults because the vaccine provides strong and long-lasting protection against herpes zoster [146]. Periodic safety reviews for other adjuvanted vaccines have repeatedly concluded that the benefits outweigh potential risks in recommended populations.

### 5.4. Implications for Lifespan Vaccination Decisions

Age-related differences in reactogenicity and benefit–risk profiles are important for vaccine policy and clinical communication. In pediatric vaccination programs, mild local and systemic reactions following adjuvanted influenza vaccines or alum-adjuvanted hepatitis B vaccines should be explained alongside their very high rates of protective immunity and their strong protection against severe disease. During pregnancy, vaccination recommendations focus on vaccines with well-established maternal and neonatal safety data, particularly influenza vaccines and tetanus-containing formulations. These vaccines help reduce maternal illness and also provide passive protection for infants through transplacental antibody transfer. For older adults, discussions often focus on the balance between increased short-term reactogenicity and improved protection against serious outcomes. The AS01-adjuvanted recombinant zoster vaccine and adjuvanted influenza vaccines are more likely to cause temporary fatigue, muscle pain, or injection-site discomfort than non-adjuvanted vaccines. However, they provide substantially stronger and longer lasting protection against herpes zoster and complications related to influenza. Clear explanation of expected side effects, their usual timing and duration, and the magnitude of clinical benefit can help maintain public confidence and support informed vaccination decisions across all age groups.

## 6. Translational and Regulatory Pathways for Next-Generation Viral Vaccine Adjuvants Targeting Durable Immunity

Advances in systems vaccinology and human-centered experimental models are changing how new vaccine adjuvants move from early discovery into clinical development. Traditional research pipelines rely heavily on mouse models. These models often fail to capture key features of human immune development, contributing to high failure rates when candidate vaccines reach human trials. For this reason, many current translational strategies emphasize approaches that are more directly relevant to human biology. These include ex vivo studies using primary human immune cells, multi-omics analysis of immune responses, and biomarker discovery programs aimed at predicting immunogenicity and long-term protection in specific populations [147]. At the same time, regulatory initiatives focused on new approach methodologies are creating general frameworks for integrating human cell-based systems, organoids, microphysiological platforms, and computational models into biomedical development. The goal is to increase the relevance of preclinical testing to human biology while also reducing dependence on animal experiments [103].

### 6.1. Human-Centric Preclinical Evaluation

Human-based experimental systems are increasingly used to screen and prioritize candidate adjuvants before clinical trials begin. Ex vivo assays using peripheral blood mononuclear cells (PBMCs) or dendritic cells from donors of different ages allow researchers to study how innate immune responses vary across the lifespan. For example, neonatal immune cells often produce lower levels of IL-12 and type I interferons and higher levels of IL-10 compared with adult cells. Older adults may also show distinct cytokine patterns. These differences influence how the immune system responds to vaccine adjuvants. Researchers have used these models to study both single adjuvants and combinations of innate immune agonists, including ligands that stimulate TLRs such as TLR7, TLR8, and TLR9. Some combinations appear to restore stronger Th1-oriented cytokine responses in neonatal immune cells, suggesting potential strategies to improve vaccine performance early in life. Similar approaches help identify age-specific patterns of synergy among different adjuvants [148,149]. These ex vivo systems are complemented by systems vaccinology analyses conducted in clinical vaccine studies. Investigators examine early gene expression patterns in blood samples collected shortly after vaccination. In several vaccines, including influenza, yellow fever, and zoster, certain transcriptional signatures measured within the first week after vaccination correlate with later antibody responses and, in some cases, with the persistence of immunity. Organ-on-chip platforms and computational models are also being explored in drug development, although their direct use in vaccine adjuvant research is still limited. At present, they mainly serve as exploratory tools that help generate hypotheses about dose selection, safety signals, or candidate prioritization, rather than acting as independent decision systems [103].

### 6.2. Emerging Adjuvants in Translational Development

Adjuvants like cyclic dinucleotide molecules activate the STING signaling pathway. These compounds show strong immune-stimulating properties in animal studies. However, translation into human vaccines has progressed more slowly. Differences among species in the cGAS-STING pathway, challenges related to drug delivery, and signals of dose-limiting toxicity have complicated development. As of 2026, human data on STING-based vaccine adjuvants remain limited to early-phase trials, and these compounds have not yet been incorporated into widely licensed viral vaccines [150,151]. For both small-molecule TLR7 or TLR8 agonists and STING pathway activators, further research in age-specific human populations will be necessary to determine whether strong innate activation in preclinical systems translates into durable, safe immune protection. This question is especially important for populations such as newborns, pregnant individuals, and older adults.

### 6.3. Clinical Trial Design for Durability Endpoints

Assessing the durability of vaccine-induced immunity requires clinical trials that extend beyond short-term immunogenicity measurements. Many modern vaccine studies include age-stratified participant groups or parallel trials in younger and older adults. This design helps researchers understand how immune responses differ across the lifespan. For several adjuvanted vaccines, including recombinant zoster vaccines that contain the AS01B adjuvant and vaccines targeting respiratory syncytial virus, large phase 3 trials and follow-up studies have shown that older adults can achieve strong initial protection. In some cases, protection remains substantial for several years, although a gradual decline in immunity has been observed for certain pathogens [152]. Trials designed to study durability often include follow-up periods of two to five years or longer. Researchers collect repeated samples to measure nAbs, MBC populations, and antigen-specific T-cell responses. Techniques commonly used include multiparameter flow cytometry, ELISPOT assays, and high-throughput serologic, transcriptomic analyses, and systems vaccinology approaches. Some investigators have suggested combining multiple immunologic measures into composite metrics that summarize the durability of vaccine responses. However, no standardized or widely accepted durability index currently exists in clinical practice. Validation of such composite measures across different vaccine trials remains an active area of research rather than an established regulatory standard.

### 6.4. Regulatory Considerations

Regulatory agencies are gradually updating their evaluation frameworks to reflect the growing complexity of modern vaccines and adjuvant systems. Organizations such as the US-FDA and the European Medicines Agency increasingly review real-world effectiveness data, post-authorization safety studies, and model-informed development strategies alongside traditional clinical trial results. These additional data sources can help clarify vaccine performance after widespread use. Large systems vaccinology datasets and multi-omics analyses are recognized as valuable research tools. They can provide insight into mechanisms of immune activation and help identify promising vaccine candidates [130]. However, their direct role in regulatory approval decisions remains limited and largely exploratory. More broadly, computational approaches such as artificial intelligence (AI)-based modeling and digital twin simulations are being explored as tools that may support clinical trial design or scenario testing [153,154]. Regulatory discussions emphasize the importance of clear validation frameworks and transparent assumptions before such models can influence decision-making. At present, these technologies cannot replace evidence from human clinical trials. Instead, they function as supplementary tools that may help integrate information about age, immune phenotype, and dose–response variation during early vaccine development.

## 7. Conclusions and Future Directions

Vaccine adjuvants have progressed from empirically selected immune stimulants to well-defined components that shape the magnitude, quality, and persistence of protective immune responses. Evidence from licensed vaccines shows that appropriate adjuvant selection can improve immunogenicity and clinical effectiveness, particularly in populations with reduced baseline immunity. The immune system varies across the lifespan, with distinct features in infancy, adulthood, and older age that influence GC activity, LLPC formation, and memory T cell differentiation. These age-specific differences provide a basis for tailoring adjuvant vaccine combinations to support durable antiviral protection. In early life, adjuvants that enhance innate immune activation and promote Th1 and Tfh responses may improve vaccine performance. Maternal immunization relies on adjuvants with established safety and the ability to support efficient transplacental antibody transfer. In older adults, stronger adjuvants that stimulate innate signaling and CD4 T cell help can partly address immunosenescence and improve the durability of protection. Booster strategies that account for both pathogen evolution and age-related immune decline remain important. Overall, adjuvants are central to vaccine durability, but their use must align with both host biology and pathogen characteristics. These benefits are particularly relevant for individuals with weaker vaccine responses, including those of advanced age or with metabolic conditions.

The molecular links between early innate activation and the long-term maintenance of plasma cells and immune memory are not fully defined in humans. Systems vaccinology has identified early transcriptional signatures in blood that correlate with later immune durability. While promising, these findings require validation across platforms and populations. Further work is also needed to define how adjuvants influence antigen trafficking to lymph nodes and the structure of follicular dendritic cell networks in humans. In addition, interactions between vaccine platforms and adjuvants need closer examination, especially as nucleic acid vaccines are used alongside protein-based boosters. Future advances will depend on integrating mechanistic studies with well-designed clinical research. In parallel, emerging adjuvant strategies that combine nanoparticle or self-assembling platforms, targeted PRR agonists, and systems vaccinology-guided optimization offer opportunities to design vaccines that not only raise higher peak titers, but also extend durability, broaden variant coverage, and more precisely tune immune responses for neonates, pregnant individuals, and older adults. Trials should include diverse age groups and extended follow-up to assess changes in antibody levels, MBCs, and T cell responses over time. Advanced tools such as single-cell transcriptomics, multiparameter flow cytometry, and high dimensional serologic assays offer opportunities to identify early immune features linked to long term protection. Connecting these features to specific adjuvants and platforms may support more rational vaccine design, including dose selection and scheduling.

Optimization of vaccination strategies will also require attention to platform combinations. In this context, adjuvant choice should be considered alongside platform and schedule, particularly for vaccines that require periodic reformulation and repeat dosing, such as seasonal influenza vaccines, where adjuvants must not only enhance responses to the current strain but also support recall responses and acceptable tolerability over multiple vaccination cycles. Heterologous approaches, such as priming with nucleic acid vaccines and boosting with adjuvanted protein vaccines, can broaden nAb responses against rapidly evolving viruses and may increase response magnitude. However, data on long-term durability remain limited and vary by pathogen. Regulatory approaches are evolving to place greater emphasis on duration of protection and immune persistence in benefit–risk assessments. Long-term follow-up requirements for newer vaccines reflect this shift. At the same time, expanding global access to adjuvanted vaccines will require progress in manufacturing capacity, cost reduction, and regulatory alignment across regions.

In summary, modern adjuvants play a key role in shaping durable immune protection. By directing innate activation, GC responses, and the development of plasma cells and memory lymphocytes, they extend vaccine effectiveness beyond the initial response. These effects are especially important in populations with age-related or disease-related immune constraints. Continued integration of mechanistic immunology, systems-level analysis, and rigorous clinical studies will be essential for developing vaccines that provide sustained protection against viral diseases across the human lifespan.

## Figures and Tables

**Table 1:** Representative viral and viral-associated vaccines administered across the human lifespan and their principal adjuvant systems.

No.	Life Stage	Vaccine	Target Pathogen/Disease	Vaccine Platform	Principle Adjuvant or Immunostimulatory Component
1	Infancy and early childhood	Hepatitis B (Engerix-B, Recombivax HB)	Hepatitis B virus	Recombinant protein	Aluminum salts (alum)
		DTaP-containing pediatric combinations	Diphtheria, tetanus, pertussis	Toxoid/subunit	Alum
		Hib conjugate vaccines	*Haemophilus influenzae* type b	Conjugate	Alum in some formulations
		Pneumococcal conjugate vaccines (PCV13/15/20)	*Streptococcus pneumoniae*	Conjugate	Aluminum phosphate in some formulations
		Inactivated poliovirus vaccine (IPV)	Poliovirus	Inactivated whole virus	None
		Inactivated influenza vaccines (pediatric formulations)	Influenza A/B	Inactivated split/subunit	Mostly none in standard pediatric formulations
		COVID-19 mRNA vaccines	SARS-CoV-2	mRNA-lipid nanoparticle	Lipid nanoparticle with intrinsic innate immune stimulation
		Rotavirus vaccines	Rotavirus	Live attenuated	None
		Measles–mumps–rubella (MMR)	Measles, mumps, rubella viruses	Live attenuated	None
		Varicella vaccine	Varicella-zoster virus	Live attenuated	None
2	Adolescence and young adulthood	Human papillomavirus vaccines (Gardasil 9, Cervarix)	HPV-associated cancers	Virus-like particle	AAHS/alum (Gardasil 9); AS04 = MPL + alum (Cervarix)
		Tdap booster	Pertussis and toxoid booster protection	Acellular subunit/toxoid	Alum
		Meningococcal conjugate vaccines	*Neisseria meningitidis*	Conjugate	Often none or alum-containing depending on product
3	Pregnancy	Maternal Tdap	Pertussis prevention in infants	Acellular subunit/toxoid	Alum
		Seasonal influenza vaccines	Influenza A/B	Inactivated split/subunit	Standard formulations usually non-adjuvanted; MF59-adjuvanted formulations generally reserved for older adults
		Maternal RSVpreF vaccine (Abrysvo)	Respiratory syncytial virus	Recombinant prefusion F protein	None
		COVID-19 vaccines	SARS-CoV-2	mRNA or protein-based	Lipid nanoparticle innate stimulation (mRNA); Matrix-M in protein nanoparticle vaccines
4	Older adulthood and immunosenescence	High-dose or adjuvanted influenza vaccines (Fluad)	Influenza A/B	Inactivated subunit	MF59 (squalene oil-in-water emulsion)
		Recombinant zoster vaccine (Shingrix)	Varicella-zoster virus reactivation	Recombinant glycoprotein E	AS01B (MPL + QS-21 liposome system)
		RSV vaccines for older adults (Arexvy, mResvia)	Respiratory syncytial virus	Recombinant protein or mRNA	AS01E in Arexvy; lipid nanoparticle platform in mRNA vaccines
		Pneumococcal vaccines (PCV20/21)	Pneumococcal disease	Conjugate	Aluminum phosphate in some formulations
		COVID-19 updated boosters	SARS-CoV-2	mRNA/protein-based	Lipid nanoparticle innate stimulation or Matrix-M
		Hepatitis B vaccines for older/high-risk adults (Heplisav-B)	Hepatitis B virus	Recombinant protein	CpG 1018 (TLR9 agonist)

**Table 2:** Representative Viral Vaccine Adjuvants and Evidence for Durability.

No.	Adjuvant	Innate Pathway(s)	Viral Vaccine(s)	Advantages	Key Limitation
1	AS01 [3,82]	TLR4, saponin-mediated inflammasome	Shingrix (VZV), RSV	Strong Th1-biased CD4^+^ responses; robust memory B-cell support; long-term efficacy in older adults	Higher reactogenicity, limited pediatric data
2	MF59 (squalene oil-in-water emulsion) [54,97]	Myeloid cell recruitment, monocyte/DC activation, chemokine and cytokine induction	Seasonal influenza	Dose-sparing; broader strain coverage and improved effectiveness versus non-adjuvanted formulations	Limited cellular durability data in older adults
3	AS03 (squalene oil-in-water emulsion with α-tocopherol) [85,86,87]	Myeloid activation, TLR-independent NF-κB activation, cytokine and chemokine induction	Pandemic influenza H1N1/H7N9	Dose-sparing; rapid induction of high titers during pandemics	Inconsistent long-term follow-up; inflammation-related AEs
4	CpG 1018 or related CpG ODNs [89,90,91]	TLR9 agonist	HepB, experimental RSV	Potent Th1-skewing adjuvant; improved seroprotection in hyporesponsive or high-risk adult populations	Limited long-term human follow-up in infants
5	Advax (delta inulin) [98,99]	Inflammasome-independent, complement activation	Influenza, SARS-CoV-2 (preclinical & clinical)	Good tolerability; promising durability and dose-sparing in preclinical models and early-phase trials	Mechanistic diversity by age; pediatric human data limited
6	STING agonists [100,101]	STING–IRF3–type I IFN	Preclinical viral vaccines (influenza, RSV)	Strong CD8^+^ T cell and Th1 responses; potential for robust antiviral and antitumor immunity	Mostly preclinical, delivery/formulation challenges
7	Nanoparticle-based TLR7/8 [98,102]	Endosomal TLR7/8 activation	Influenza, SARS-CoV-2 (preclinical)	Targeted delivery to APCs; strong GC and Tfh responses at low doses	Human durability data lacking; manufacturing scale-up

## Data Availability

No new data were created or analyzed in this study. Data sharing is not applicable to this article.
